# Actin Depolymerizing Factors Cofilin1 and Destrin Are Required for Ureteric Bud Branching Morphogenesis

**DOI:** 10.1371/journal.pgen.1001176

**Published:** 2010-10-28

**Authors:** Satu Kuure, Cristina Cebrian, Quentin Machingo, Benson C. Lu, Xuan Chi, Deborah Hyink, Vivette D'Agati, Christine Gurniak, Walter Witke, Frank Costantini

**Affiliations:** 1 Department of Genetics and Development, Columbia University Medical Center, New York, New York, United States of America; 2 Department of Medicine, Mount Sinai School of Medicine, New York, New York, United States of America; 3 Department of Pathology, Columbia University Medical Center, New York, New York, United States of America; 4 Institute of Genetics, University of Bonn, Bonn, Germany; Duke University, United States of America

## Abstract

The actin depolymerizing factors (ADFs) play important roles in several cellular processes that require cytoskeletal rearrangements, such as cell migration, but little is known about the *in vivo* functions of ADFs in developmental events like branching morphogenesis. While the molecular control of ureteric bud (UB) branching during kidney development has been extensively studied, the detailed cellular events underlying this process remain poorly understood. To gain insight into the role of actin cytoskeletal dynamics during renal branching morphogenesis, we studied the functional requirements for the closely related ADFs cofilin1 (*Cfl1*) and destrin (*Dstn*) during mouse development. Either deletion of *Cfl1* in UB epithelium or an inactivating mutation in *Dstn* has no effect on renal morphogenesis, but simultaneous lack of both genes arrests branching morphogenesis at an early stage, revealing considerable functional overlap between cofilin1 and destrin. Lack of *Cfl1* and *Dstn* in the UB causes accumulation of filamentous actin, disruption of normal epithelial organization, and defects in cell migration. Animals with less severe combinations of mutant *Cfl1* and *Dstn* alleles, which retain one wild-type *Cfl1* or *Dstn* allele, display abnormalities including ureter duplication, renal hypoplasia, and abnormal kidney shape. The results indicate that ADF activity, provided by either cofilin1 or destrin, is essential in UB epithelial cells for normal growth and branching.

## Author Summary

Development of the ureter and collecting ducts of the kidney requires extensive growth and branching of an epithelial tube, the ureteric bud. While many genes that control this process are known, the cellular events that underlie renal morphogenesis remain poorly understood. Many cellular changes that might contribute to ureteric bud morphogenesis, such as migration and changes in shape, involve the actin cytoskeleton. Actin depolymerizing factors (ADFs) are important for changes in the organization of the cytoskeleton in cultured cells, but the roles of the ADF genes *in vivo* remain to be fully elucidated. Here, we examine the importance of the ADFs cofilin1 and destrin in ureteric bud branching and find that lack of both genes arrests this process at an early stage, while lesser reductions in ADF gene dosage cause more subtle defects in kidney development. This finding may help us to understand the origins of certain congenital malformations in humans.

## Introduction

Depolymerization and severing of actin filaments produces new actin monomers and new free ends that facilitate dynamic changes in the actin cytoskeleton. These events are essential for several cellular processes including cell survival, shaping, cytokinesis, migration and chemotaxis [1]. For example, during migration and chemotaxis, cell protrusions are formed as a result of localized actin polymerization in the leading edge of a motile cell [2]. In dividing cells, actin depolymerization plays an important role in chromosome congression, cleavage plane orientation and furrow formation [3]. Three genes encode actin depolymerization factors (ADFs) in mammals: *Cofilin1* (*Cfl1,* non-muscle Cofilin, n-Cofilin), *Cofilin2* (*Cfl2*, muscle Cofilin) and *Destrin* (*Dstn*, also called *ADF* or *Corn1*).

Despite the vast amount of *in vitro* data on the functions of ADFs, remarkably little is known about their *in vivo* roles. ADF genes share overlapping expression patterns in many cell types, but the phenotypes of mouse mutants in either *Cfl1* or *Dstn* suggest that they have somewhat distinct *in vivo* functions. Mice lacking *Cfl1* are embryonic lethal at E11.5–12.5 and display defects in neural tube closure and neural crest cell migration [4], even though *Dstn* is highly expressed in the cranial neuroectoderm of these mutant embryos; thus, in this situation, *Dstn* seemed unable to compensate for the absence of *Cfl1*. No *in vivo* data on *Cfl2* function are yet available. *Dstn* ^−/−^ homozygotes are viable but have corneal defects leading to eventual blindness in adult mice, whereas two alleles with inactivating point mutations (*Dstn^corn1^* and *Dstn^corn1-2J^*) cause the same phenotype, indicating that they behave as null alleles [5], [6]. While *Dstn^−/−^* brains have a normal gross morphology, conditional deletion of *Cfl1* in neuronal cells causes excessive differentiation, changes in cell proliferation, and migration defects, resulting in a lissencephaly phenotype [5].

While *Cfl1* seems to be essential for the normal development of the neural tube and neuronal differentiation, the requirement for specific ADFs in other basic morphogenetic processes has not been addressed. Several organs including mammary and salivary gland, lung and kidney develop largely from an epithelial outgrowth that subsequently branches in an organ-specific manner. Kidney development, which serves as an excellent model to study epithelial branching, begins by the formation of the ureteric bud (UB) (at E10.5), which first arises as a thickening of the Wolffian (or nephric) duct. Subsequently, the UB elongates, invades the adjacent metanephric mesenchyme (MM) and begins to branch in a repeated and characteristic pattern, a process that continues until early postnatal life, and gives rise to the tree-like collecting duct system. The UB branching rate and pattern are tightly regulated by signals from the surrounding MM, while at the same time, the UB tips induce nephron progenitors in the MM to undergo mesenchyme-to-epithelium transformation, leading to formation of different nephron segments [7]–[9].

We became interested in potential role of ADF genes in renal epithelial branching for several reasons. First, evagination of the UB from the WD and its subsequent growth and branching require a number of cellular processes that involve the actin cytoskeleton, such as cell migration and proliferation [10], [11]. *In vitro* inhibition of ROCK, an upstream regulator of Cofilin1 activity, has controversial effects on renal development as it can either increase or decrease kidney size [12], [13]. Second, it was reported that *Cfl1* gene expression is upregulated by the expression of activated forms of the Ret receptor tyrosine kinase in NIH3T3 cells [14]. Ret, which is expressed by UB cells, is the receptor for the secreted protein GDNF, which is produced by the MM. Ret and GDNF (as well as the GDNF co-receptor Gfrα1) play a critical role in UB branching morphogenesis, and their absence leads to renal agenesis in mice and humans [15]–[17].

Here we show that mice lacking *Cfl1* in the ureteric epithelium, or those with an inactivating mutation in *Dstn*, develop mostly normal kidneys. However, simultaneous inactivation of both genes in UB epithelium causes a severe and early block in UB branching, indicating considerable functional overlap between cofilin1 and destrin in UB cells. Double mutant UB epithelial cells accumulate excess filamentous actin (F-actin), resulting in irregular epithelial organization and a defect in cell migration. The results show that actin depolymerization by either *Cfl1* or *Dstn* is required for normal growth and branching of the UB epithelium during renal branching morphogenesis.

## Results

### Abnormal ureteric budding and kidney shape in *Cfl1^+/−^;Dstn^−/−^* compound mutants

**Figure 1:**
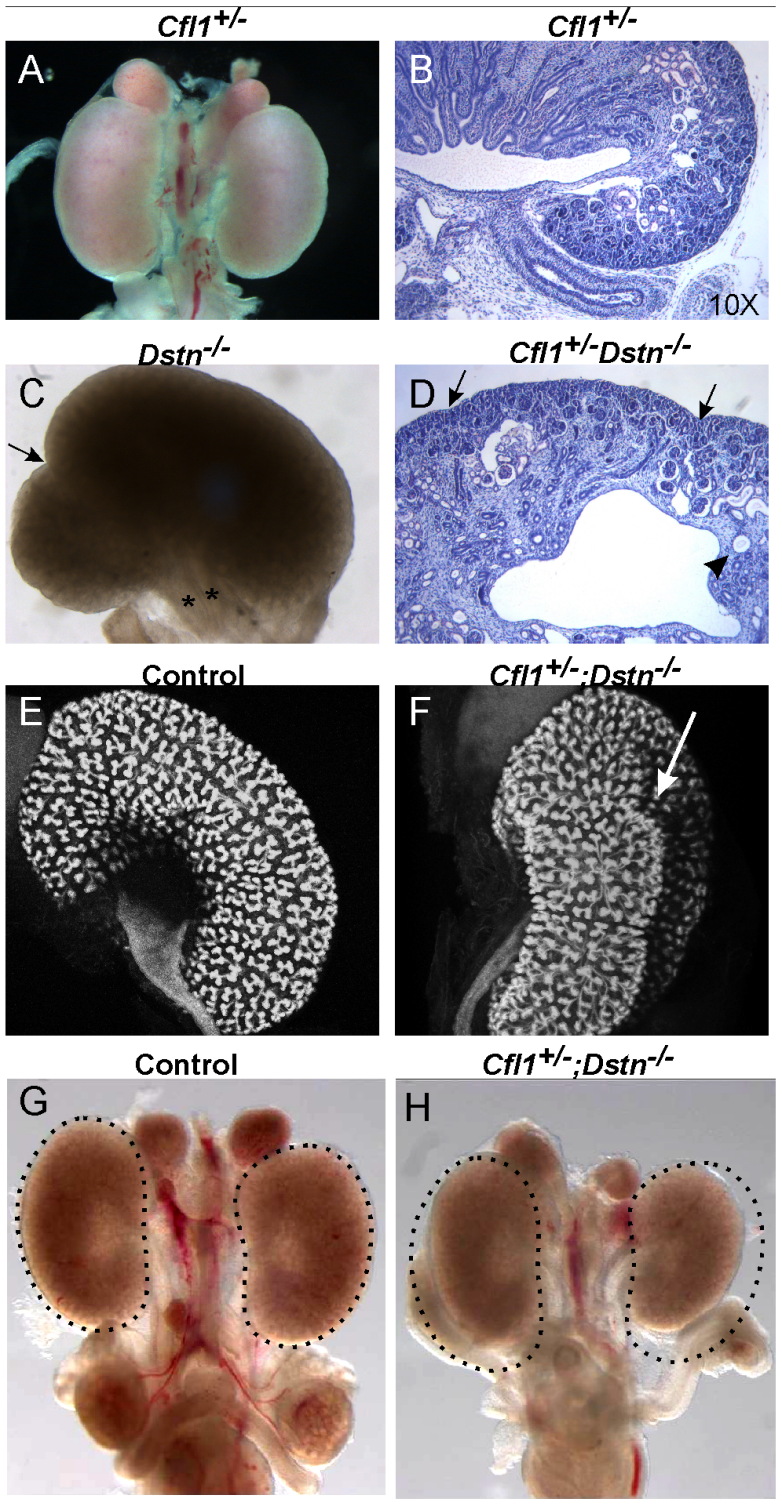
Kidney defects in *Dstn^−/−^* and *Cfl1^+/−^*;*Dstn^−/−^* mutant animals. Whole mount image (A) and histological section (B) of control kidneys. (C) Double ureter (asterisks) and duplex kidney formation in a *Dstn^−/−^* embryo. Arrow points to the cleft between the two lobes of the duplex kidney, and asterisks mark the two ureters. (D) Histology of *Cfl1^+/−^*;*Dstn^−/−^* kidney reveals irregular surface of the renal cortex. Arrows point to constriction sites, arrowhead to a dilated collecting duct. (E) and (F), Three-dimensional reconstruction from confocal optical sections of control kidney (*Hoxb7/myrVenus;Cfl1^+/−^*) (E), showing smooth surface and kidney shape, and *Hoxb7/myrVenus;Cfl1^+/−^;Dstn^−/−^* kidney (F), showing an irregular surface and abnormal cleft (arrow). (G) and (H), reduced kidney size in *Cfl1^+/−^;Dstn^−/−^* (H) compared to *Dstn^+/−^* control (G). The dotted curves indicate the outlines of the two control kidneys. All kidneys are at E16.5, except C which is E17.5.

**Figure 2:**
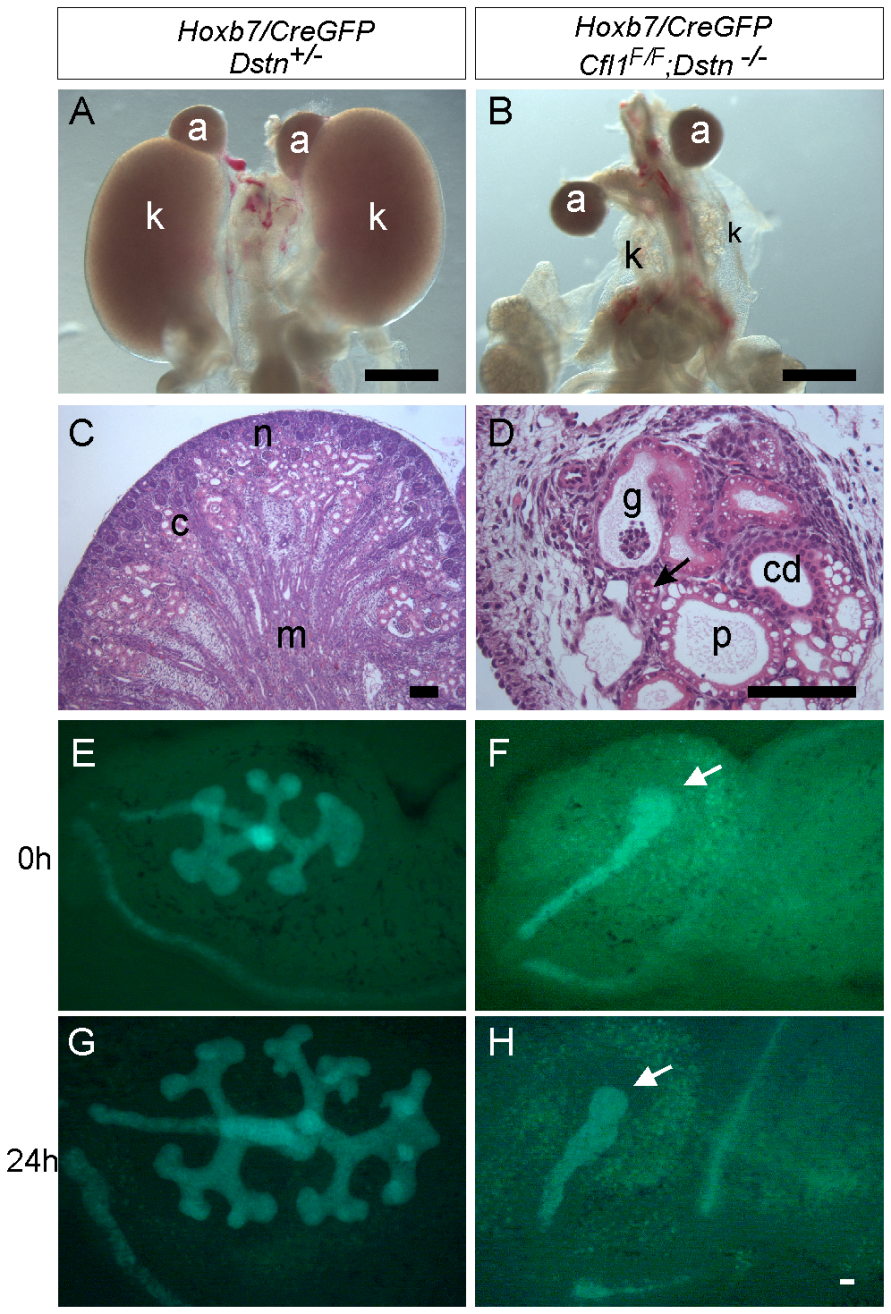
Absence of *Cfl1* and *Dstn* in the ureteric bud of embryonic kidneys results in severe renal hypodysplasia. (A) Control kidneys at E18.5. (B) Absence of normal kidneys in E18.5 double mutant where two *Cfl1^F/F^* alleles are conditionally deleted by *HoxB7/CreGFP,* specifically in ureteric epithelium, in a *Dstn ^−/−^* background. (C) and (D), Hematoxylin-eosin staining of E18.5 control kidney (C) and *Cfl1;Dstn* double mutant kidney (D). Aside from their very small size, the mutant kidneys lack normal patterning, and contain only a few disorganized tubules. There are a few nephrons containing proximal tubules (p) and distal tubules (arrow), and very few tubules resembling collecting ducts (cd). (E–F) ureteric bud branching in double mutant and control kidneys. Control *Dstn* ^+/−^ kidneys show normal branched ureteric bud morphology at E12.5 (E) and continue to branch when cultured for 24 hours (G). In contrast, *Cfl1;Dst*n double mutants show an unbranched ureteric bud outgrowth (white arrow) at E12 (F), which fails to branch further when cultured for 24 hours (H). Abbreviations: a, adrenal gland; c, cortex; g, glomerulus; k, kidney; m, medulla; n, nephrogenic zone; Scale bar 1 mm for A–B; 200 µm for C–H 100 µm.

*Cfl1* and *Dstn* are both expressed in most or all cells of the developing kidney, while *Cfl2* is apparently not expressed in kidney [18]. In order to investigate the requirement for ADF activity in ureteric bud morphogenesis, we studied the effects *Dstn* and *Cfl1* mutations during renal development, initially using conventional loss-of function alleles. For *Dstn*, we used the *Dstn^corn1-2J^* mutant allele, which is phenotypically similar to the knockout allele [5], [6] and we therefore refer to *Dstn^corn1-2J^* heterozygotes and homozygotes as *Dstn* ^+/−^ and *Dstn^−/−^*. We observed no renal or ureteric abnormalities in *Cfl1* ^+/−^ or *Dstn* ^+/−^ heterozygotes (Figure 1A and 1B, and Figure 2A, 2C, 2E, 2G), or in *Dstn^+/−^;Cfl1^+/−^* compound heterozygotes (Table 1, column 3). Twenty-three percent of *Dstn^−/−^* embryos displayed a duplicated ureter, sometimes resulting in a duplex kidney (Table 1, column 1; Figure 1C, Figure S1A, S1B), but renal development appeared otherwise normal (data not shown). Surprisingly, none of the twenty-one *Cfl1^+/−^*;*Dstn^−/−^* embryos we examined displayed ureter duplications (Table 1, column 4), but instead 38% had kidneys that were abnormally shaped, elongated and uneven on the surface (Figure 1D, 1F), while an additional 29% had mildly hypoplastic kidneys (approximately 25% reduced in size, e.g., Figure 1H). Cultures of E12.5 *Cfl1^+/−^*;*Dstn^−/−^* renal explants sometimes showed a slight delay in branching, resulting in reduced UB tip numbers, but no obvious branching pattern abnormalities that might explain the abnormal kidney shapes were observed (Figure S1C, S1D, S1E, S1F). It is not clear why removing one *Cfl1* allele would apparently rescue the ureteric duplications seen in some *Dstn−/−* mutants, but this may be a consequence of the mixed genetic background. Overall, however, the spectrum of ureteric and mild renal abnormalities observed in some *Dstn* ^−/−^ and *Cfl1^+/−^*;*Dstn^−/−^* kidneys suggests a role for actin depolymerization during renal branching morphogenesis.

**Table 1:** Frequency of ureter and kidney defects in mice with two or three mutant alleles.

Column:	1	2	3	4	5	6	7	8
*Dstn* genotype	*−/−*	*−/−*	*+/−*	*−/−*	*+/−*	*−/−*	*+/−*	*+/−*
*Cfl1* genotype	*+/+*	*+/+*	*+/−*	*+/−*	*Flox/+*	*Flox/+*	*Flox/−*	*Flox/Flox*
Other alleles		*Cxcr4^−/−^*			*Cre*	*Cre*	*Cre*	*Cre*
N =	35	7	12	21	6	4	7	4

Phenotypes								
double ureter	23%	28%	nd	nd	nd	50%	14%	25%
misshapen kidney	nd	nd	nd	38%	nd	nd	nd	25%
hypoplastic kidney	nd	nd	nd	29%	nd	nd	nd	27%

N: numbers of mice analyzed. nd: not detected. *Cre*: *Hoxb7/CreGFP*.

### The gene dosage of *Cfl1* and *Dstn* in the ureteric bud is important for normal branching patterns

Since *Cfl1* and *Dstn* are expressed in most or all cell types in the kidney, the abnormal renal morphogenesis in some *Cfl1^+/−^; Dstn^−/−^* compound mutants could be due to either a direct effect in ureteric bud cells, or an indirect effect of faulty induction by the MM or stroma. To address this issue, and also to circumvent the early lethality in *Cfl1* ^−/−^ mice [4] we used a conditional knockout strategy to delete one or both *Cfl1* alleles in the Wolffian duct and ureteric bud lineage. Mice carrying a floxed *Cfl1* allele, *Cfl1^F^* [5] were crossed with *Hoxb7/CreGFP,* a transgenic line expressing Cre recombinase together with GFP in the Wolffian duct (from ∼E9.5) and UB [19]. Deletion of one or both *Cfl1* alleles in a *Dstn* ^+/+^ background (*Hoxb7/CreGFP;Cfl1^F/F^* or *Hoxb7/CreGFP; Cfl1^F/+^*) had no apparent effect on ureter or kidney development (data not shown). This suggested that *Cfl1* is not required in the WD/UB lineage when *Dstn* is present at wild-type levels. However, deleting both *Cfl1* alleles in the UB in a *Dstn* ^+/−^ background (Table 1, column 8), or deleting one *Cfl1* allele in the UB in a *Dstn* ^−/−^ background (Table 1, column 6), caused occasional ureter duplications, and the same occasional renal hypoplasia or abnormal kidney shapes as seen in *Cfl1^+/−^;Dstn^−/−^* mice (Table 1, column 4). While the low frequency of animals with each of these specific genotypes precluded a quantitative comparison, the combined results indicate that normal cofilin1 levels in the UB are important, when *Dstn* is either reduced or absent, for normal renal and ureteric morphogenesis.

### Homozygous deletion of *Cfl1* in ureteric epithelium, in the absence of *Dstn*, abrogates ureteric bud branching and results in severe renal hypodysplasia

When both *Cfl1* alleles were deleted in the UB, in the *Dstn^−/−^* background (*Hoxb7/CreGFP; Cfl1^F/F^; Dstn^−/−^*) kidney development failed almost completely (Figure 2A and 2B). Histological analysis revealed the presence of ureter and different nephron segments (glomeruli, proximal and distal tubules) but very little collecting duct epithelium (Figure 2D), and no renal pelvis or proper cortex-medulla compartmentalization. *Hoxb7/CreGFP; Cfl1^F/−^; Dstn^−/−^* kidneys (with one null and one floxed *Cfl1* allele) appeared identical to *Cfl1^F/F^; Dstn^−/−^* (data not shown), and both of these genotypes will hereafter be called “double mutant”.

As double mutant kidneys contained virtually no UB derivatives at E18 (Figure 2D), we wanted to study the development of the UB at earlier stages. We used the GFP encoded by the *Hoxb7/CreGFP* transgene to visualize the UB in control and double mutant kidneys. The ureteric bud normally grows out from Wolffian duct at E10.5, by E11.5 it has branched once to form the “T-bud” shaped kidney, and by E12.5 it has branched several more times (Figure 2E). In all double mutant embryos examined at E11.5 (N = 6) or E12.5 (N = 8), the two UBs had successfully grown out from the Wolffian ducts and elongated, but none of them had branched within the kidney (Figure 2F). When cultured for 24 hours, the control kidneys continued to branch (Figure 2G), while the double mutant failed to branch (Figure 2H). Thus, deletion of *Cfl1* using *Hoxb7/CreGFP*, in the absence of *Dstn*, did not prevent UB outgrowth, but completely blocked subsequent branching.

**Figure 3:**
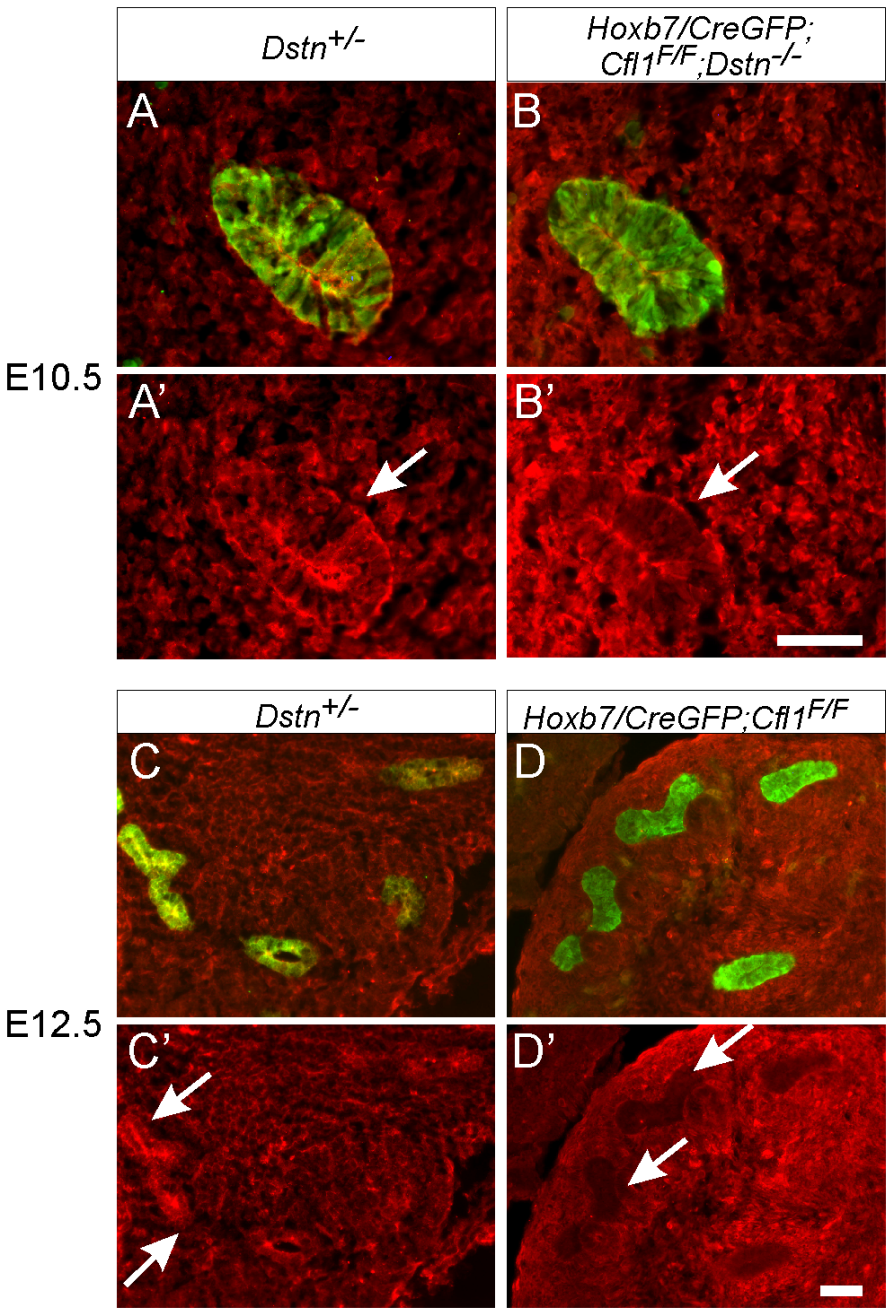
Efficiency of *Hoxb7/CreGFP*-mediated deletion of *Cfl1* as evaluated by Cofilin1 antibody staining. Calbindin (green) was used to visualize Wolffian duct epithelium in (A–D). (A–B'), cofilin1 (red) and calbindin (green) in E10.5 Wolffian duct (sections through the expanded region that will give rise to the UB). Cofilin1 is expressed in every cell in both ureteric bud and metanephric mesenchyme, and seems slightly enriched in UB epithelial cells. At E10.5, there is no detectable difference in cofilin1 protein amount or localization between control (*Dstn^+/−^*) and *Hoxb7/CreGFP; Cfl1^F/F^; Dstn^−/−^* kidneys. (C–D'), At E12.5, cofilin1 localization in the *Dstn^+/−^* control is similar to that at the earlier stages. However, *Hoxb7/CreGFP* has efficiently deleted the gene in the UB, as the protein is absent in ureteric epithelium of *Cfl1^F/F^* kidneys. Arrows point to ureteric buds, scale bar 50 µm.

The finding that the UB always formed in *Cfl1*;*Dstn* double mutant kidneys, but failed to branch, raised the possibility that the activities of cofilin1 and destrin are required for UB branching, but not for Wolffian duct growth or initial UB formation. Alternatively, there might be a delay in the elimination of cofilin1 by *Hoxb7/CreGFP,* such that there is still sufficient cofilin1 at the time of UB outgrowth (E10.5) but not when branching initiates (E11.5). To study the timing and efficiency of cofilin1 elimination we stained mutant kidneys with anti-cofilin1 antibody. While the UB epithelium was clearly devoid of cofilin1 at E11.5 (data not shown) and E12.5 (Figure 3C–3D'), confirming the activity of *Hoxb7/CreGFP*, we found that cofilin1 protein levels at E10.5 were normal in the forming ureteric bud (Figure 3A–3B'). Thus, the ability of the UB to grow out in double mutants, but not to branch subsequently, is most likely due to residual cofilin1 expression at E10.5, which is eliminated by E11.5. A similar delay in cofilin1 protein elimination was observed when the floxed *Cfl1* allele was deleted in the brain using *nestin^Cre^*, a result that was attributed to the long half-life of the protein [5].

### Exogenous GDNF is unable to rescue ureteric branching in *Cfl1*;*Dstn* double mutant kidneys

**Figure 4:**
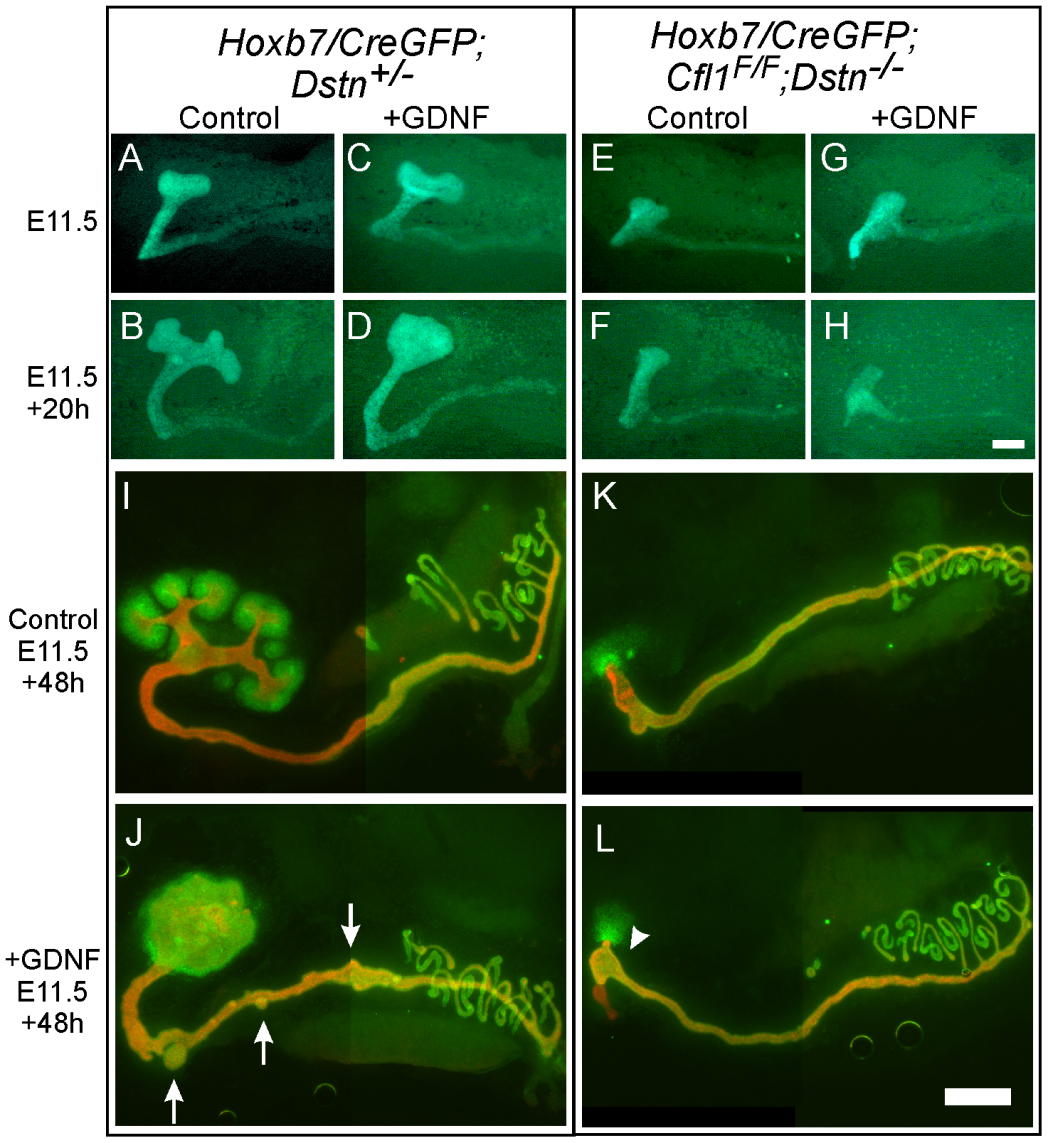
Exogenous GDNF is unable to rescue the ureteric bud branching defect, or induce ectopic budding from Wolffian duct, in *Cfl1;Dstn* double mutants. (A–H) Time-lapse images of E11.5 kidney cultures in control medium (A, B, E, F) or with added GDNF (C, D, G, H). Control kidneys start from early T-shape (A, C) and without GDNF end up with secondary branch points at 20 h (B), but when cultured for 20 h with exogenous GDNF, they develop a huge enlargement of the ureteric bud tip (D). *Cfl1;Dstn* double mutant kidneys show only a small, unbranched ureteric bud at E11.5 (E, G), which during 20 hrs of culture is able to elongate but does not begin branching regardless of GDNF addition (F, H). (I–L), E11.5 urogenital blocks (UGBs) cultured for 48 h without (I, K) or with GDNF (J, L) and stained for Pax2 (green) and pan-cytokeratin (red). Under normal conditions, the control *Dstn^+/−^* kidney (I) shows normal branching and Pax2-positive induced metanephric mesenchyme surrounding the ureteric tips, while the *Cfl1;Dstn* double mutant kidney (K) retains some Pax2-positive mesenchyme around a single outgrowth of ureteric bud. In a control *Dstn^+/−^* UGB (J), exogenous GDNF induced massive swelling of the ureteric tip, which still maintains Pax2-positive metanephric mesenchyme around it, and induced extra ureteric budding (arrows) from the Wolffian duct. GDNF is unable to induce normal ureteric bud branching or ectopic ureteric budding in *Cfl1;Dstn* double mutant UGB (L), but induces slight bulging (arrowhead) in the lower ureteric bud epithelium. Scale bar 200 µm.

GDNF/Ret signaling is one of the most important pathways that promotes primary UB formation and subsequent branching [16]. To test if the UB branching defect in *Cfl1*;*Dstn* double mutants is due to insufficient GDNF/Ret signaling (as occurs in many other mutants with defective UB branching) [8], [15], we cultured double mutant kidneys with or without exogenous GDNF. When cultured without added GDNF, control kidneys with early T-shaped UBs at E11.5 developed several secondary branches over the next 48 h in culture (Figure 4A, 4B, 4I), while those cultured with GDNF showed a swelling of the UB tip and ectopic budding from Wolffian duct (Figure 4C, 4D, 4J), the typical response [20]. The *Cfl1*;*Dstn* double mutant UBs had not branched normally when dissected at E11.5 (Figure 4E) and when cultured for 48 hrs without added GDNF they elongated slightly but did not branch (Figure 4F, 4K). Added GDNF had no effect on UB morphogenesis in *Cfl1*;*Dstn* double mutant kidneys cultured from E11.5, and it was also unable to induce ectopic ureteric bud formation from the double mutant Wolffian duct (Figure 4G, 4H, 4L). However, all kidneys lacking two or three out of the four *Cfl1*;*Dstn* alleles responded like wild-type kidneys to GDNF treatment (data not shown).

The finding that exogenous GDNF was unable to rescue UB branching in *Cfl1*;*Dstn* double mutant kidneys had at least two potential explanations. First we explored the possibility that lack of expression of the GDNF receptor Ret impairs the ability of ureteric epithelium to respond to GDNF signals. However, we found that *Ret* was expressed in a normal pattern (i.e., at the UB tip) and normal level in E11.5 double mutant kidneys (Figure S2A, S2B), but was somewhat reduced at a slightly later stage (Figure S2C, S2D). Like *Ret*, the Gdnf/Ret target gene *Wnt11* [21] was expressed normally in the E11.5 UB tip (Figure S2E, S2F). Thus, a lack of *Ret* expression or Ret signaling in the UB seemed not to be the cause of the failure to branch. We next explored an alternative explanation, that defective branching was primarily due to cytoskeletal changes caused by the lack of ADF activity.

### Lack of cofilin1 and destrin in UB epithelium causes F-actin accumulation and irregular cell shapes

**Figure 5:**
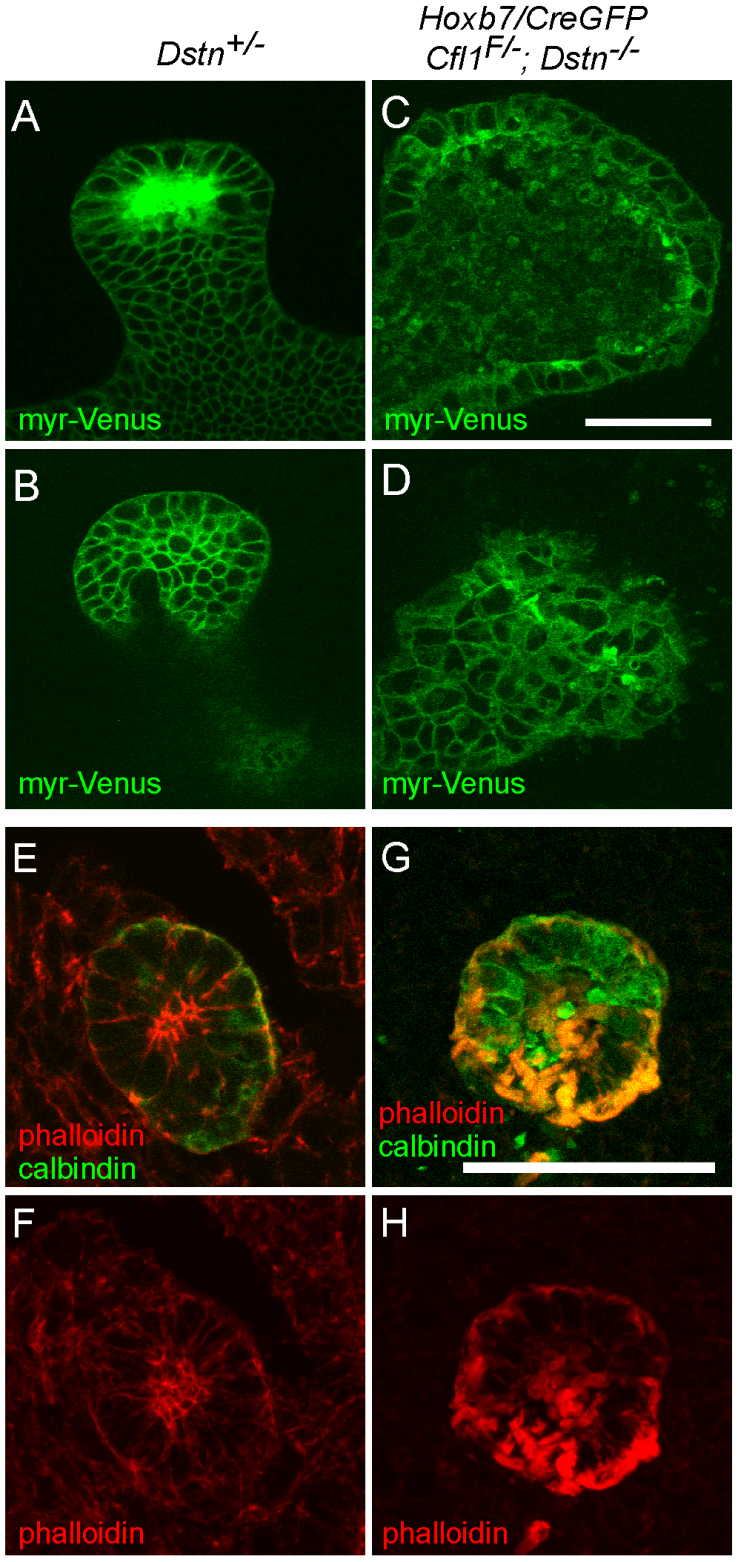
Disruption of actin depolymerization results in irregular ureteric epithelial cell shape and organization due to F-actin accumulation in *Cfl;Dstn* double mutant ureteric bud. (A–D), A transgene encoding myristoylated-Venus driven by *Hoxb7*-promoter was introduced to *Cfl1;Dstn* mutant mice to visualize ureteric epithelial cell outlines; optical sections through ureteric bud tips of control *Dstn^+/−^* (A–B) and double mutant (C–D) kidneys at E12.5. (A) and (C) are optical sections through the lumen of the UB tip, while (B) and (D) are glancing sections through the epithelium. Control ureteric epithelium shows cells that vary in shape, but have smooth outlines and are organized in an orderly pattern along the whole epithelium. (C–D), Double mutant epithelial cells are often abnormal in shape and exhibit a disorganized pattern throughout the ureteric bud. (E–H), Confocal images of phalloidin (red)/calbindin (green) double staining, which visualizes actin filaments (F-actin) in developing kidney. (E–F), In control kidney, actin filaments are enriched in apical side of ureteric epithelium while some localize to the basolateral surfaces. (G–H) Disruption of actin depolymerization in *Cfl1;Dstn* double mutants results in huge accumulation of F-actin, specifically in the ureteric epithelium where *Cfl1^F^* is deleted. Scale bars: 50 µm.

Dynamic actin cytoskeleton rearrangements are involved in several cellular processes, such as apoptosis, proliferation and migration [1]. We took the advantage of the *Hoxb7/myrVenus* transgenic line, which expresses myristylated-Venus fluorescent protein at the cell membrane [22], to visualize epithelial cell shape and organization in the ureteric buds of *Cfl1*;*Dstn* double mutant mice. Confocal scanning of the UB epithelium at E12.5 revealed a variety of cell shapes in control kidneys, but the epithelium was well organized and cell outlines smooth and distinct (Figure 5A and 5B). In contrast, UB epithelial cells in *Cfl1*;*Dstn* mutant kidneys were disorganized, irregular in size and shape, and contained abnormal membranous (i.e., Venus-positive) bodies (Figure 5C and 5D). Thus, lack of both cofilin1 and destrin in the UB disrupts normal epithelial cell shape and organization.

*In vivo*, mutations in ADF genes have variable consequences on F-actin; they can cause accumulation of actin filaments [5], [6], [23] or a shortage of actin filaments [4], [23]. The latter suggests involvement of ADFs in actin nucleation, and is supported by *in vitro* studies [24], [25]. To understand how the absence of cofilin1 and destrin in UB epithelium affects the actin cytoskeleton, we stained sections of control and double mutant kidneys with phalloidin to visualize F-actin at E11.5 (data not shown) and E12.5 (Figure 5E–5H). As reported previously [12], the strongest phalloidin staining in control kidneys was observed at apical membranes of UB epithelial cells, but maximal projections of confocal images also revealed some actin filaments at the basolateral membranes (Figure 5E–5F). In accordance with the normal cofilin1 levels in UB epithelium of *Cfl1*;*Dstn* double mutant kidneys at E10.5 (Figure 3A–3B'), phalloidin staining was indistinguishable in double mutant and control kidneys at this stage (Figure S3). However, the epithelium of double mutant kidneys was full of phalloidin-positive inclusions at E11.5 (data not shown) and E12.5 (Figure 5G–5H). Accumulation of F-actin was strongest in the apical membranes but also obvious on the basolateral sides of mutant epithelial cells. These data suggest that cofilin1 and destrin are not required for actin nucleation in the UB epithelium but pivotal in its depolymerization and turnover.

### Impaired actin depolymerization results in cell migration defect in UB epithelium of *Cfl1*;*Dstn* double mutant kidneys

Accumulation of actin filaments within the cells can impair their proliferation and migration [1]. No differences in the mitotic indices (% of phosphohistoneH3+ cells) of *Cfl1*;*Dstn* double mutant (1.6%±0.5, n = 4) and control (1.6%±0.3, n = 4) UB epithelium were detected at E11.5 (data not shown) suggesting that the primary cause for the branching defect in mutant mice is not a defect in cell proliferation. While the double mutant UBs did not branch, their continued elongation after E11.5 presumably reflects this continuing cell proliferation.

Primary cell cultures derived from the UB [26], allowed us to apply a scratch assay to non-immortalized primary cell cultures obtained from UBs of different *Cfl1;Dstn* genotypes. Briefly, individual UBs at E11.5 or E12.5 were separated from the surrounding mesenchyme and plated in fibronectin-coated wells, where the UB cells attached to the bottom and formed monolayers within the next 48 h. These primary epithelial cells survived for approximately two weeks without immortalization (for details, see Materials and Methods). The UB cells of all genotypes including double mutants remained quiescent as judged by lack of the proliferative marker Ki67 (Figure S4A and data not shown). The cells in such cultures were positive for the UB epithelial marker pan-cytokeratin, confirming their origin from the UB (Figure S4B). No differences in the capacity to adhere or form monolayers were observed between control cells (e.g., *Dstn−/−*) and those from *Cfl1*;*Dstn* double mutant UBs (Figure S4C, S4D).

**Figure 6:**
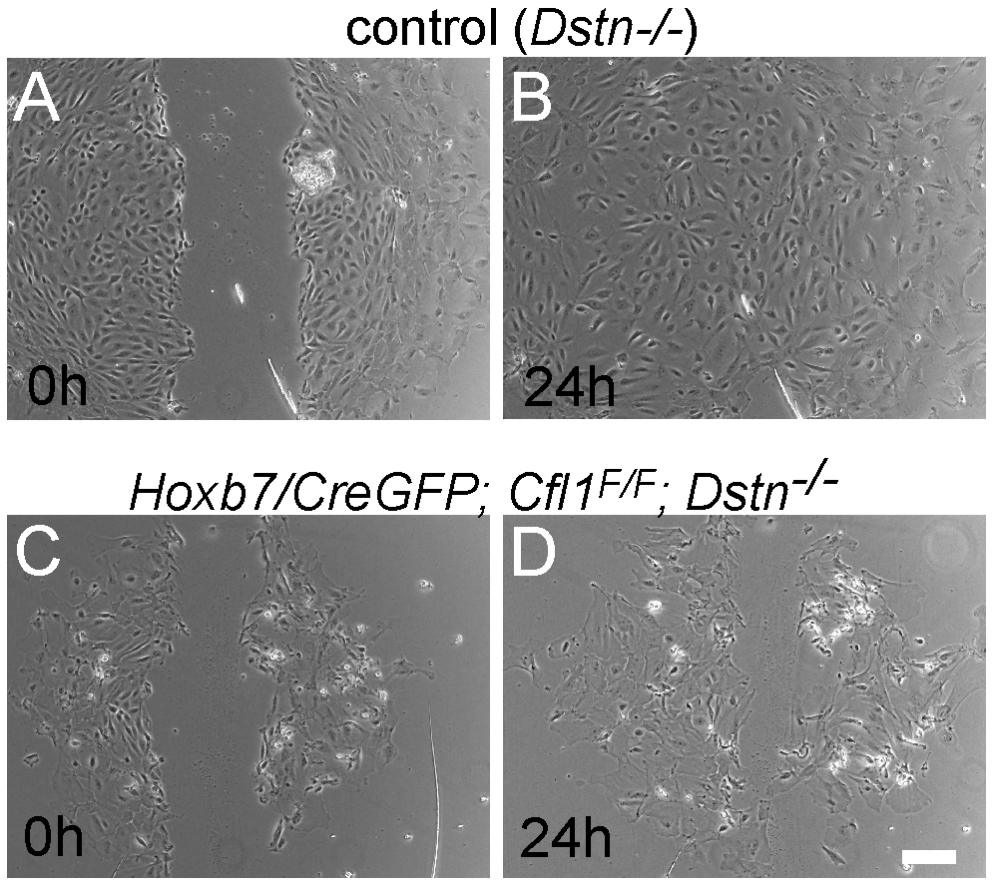
Migration assay for primary ureteric bud cells reveals defects in epithelial cell movement in *Cfl1;Dstn* double mutant. A scratch was introduced 2 days after plating the isolated ureteric buds (0 h, A and C) and followed for 24 h (B and D). Control *Dstn^−/−^*cells (A–B) had completely filled the gap produced by the scratch (N = 12 cultures), while double mutant cells (C–D) were impaired in their movement (N = 5 cultures). Scale bars 200 µm.

Migration of epithelial cells was studied by introducing a scratch in the confluent cell monolayers at 48 h after plating the UB. Cultures were photographed after 3 h, 8 h and 24 h. The control cells migrated to completely fill in the gap by 24 h (n** = **12, Figure 6A and 6B), while *Cfl1*;*Dstn* double mutant cells showed some movement but always failed to close the gap (n = 5, Figure 6C and 6D). Similarly to what we observed in the UB epithelium of E11.5 and E12.5 double mutant kidneys *in vivo*, the double mutant cells in culture were heterogeneous in size and morphology (Figure 6C and 6D) and huge F-actin accumulation was evident by phalloidin staining (data not shown), as it is in the intact UB (Figure 5).

**Figure 7:**
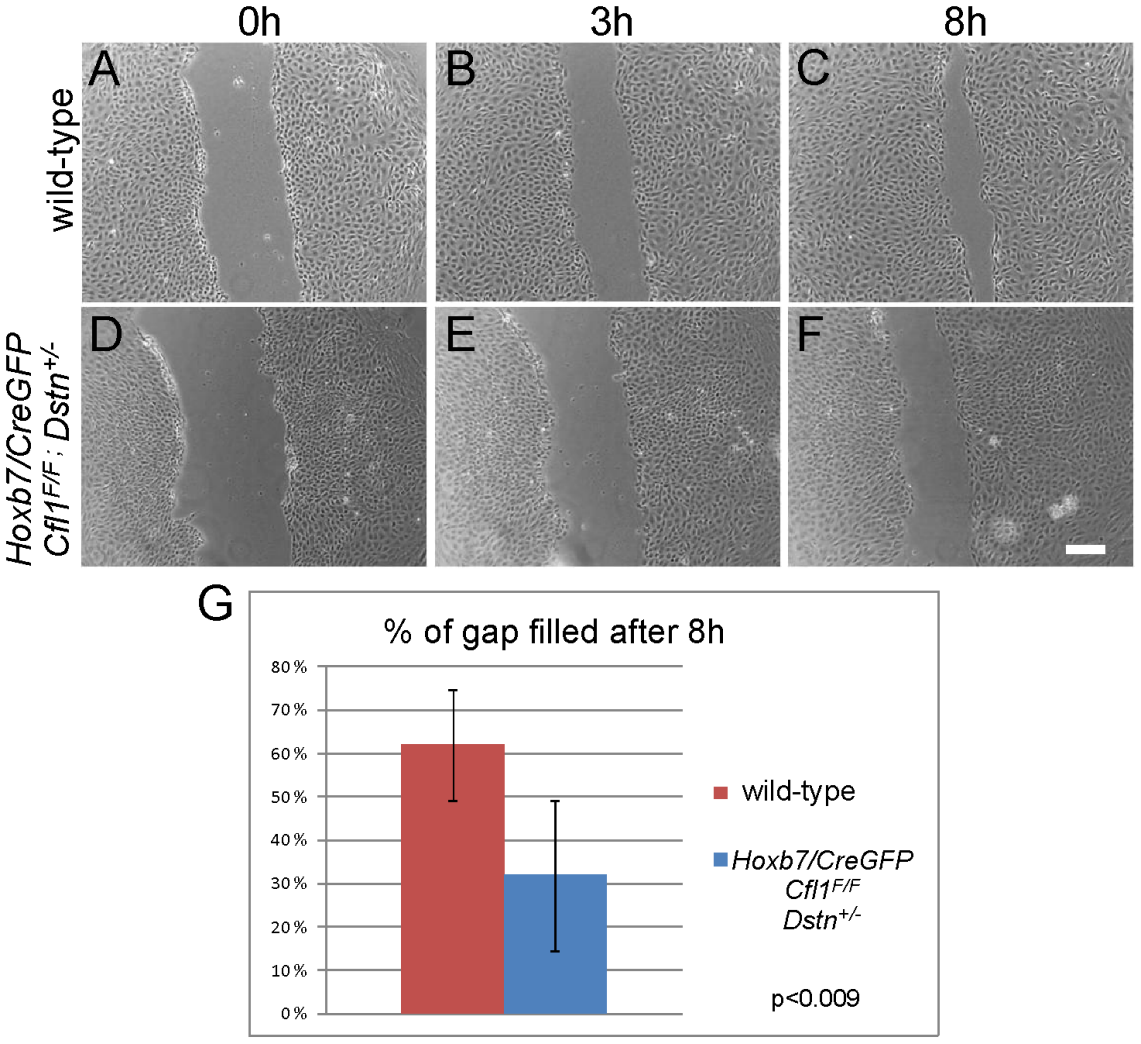
Primary UB epithelial cells lacking *Cfl1* but maintaining one wild-type *Dstn* allele (*Hoxb7/CreGFP; Cfl1^F/F^; Dstn+/−*) show a migration delay *in vitro*. Examples of wild type control (A–C) and mutant (D–F) primary ureteric bud epithelial cell migration in scratch assay. Already at 8 h after establishment of the scratch, wild-type cells (N = 12) have migrated to almost fill the gap (A–C), while mutant cells (D–F, N = 9) have not migrated as far. (G) Quantification of movements by control and mutant primary epithelial cells at 8 h after introducing the scratch. The percent of gap filled at this time point was calculated by dividing the average width of gap at 8 h by the width of initial scratch. Scale bar 200 µm.

As *Cfl1*;*Dstn* double mutant kidneys are delayed in their growth at E11.5 (Figure 4), we were concerned that, in addition to the abnormal cell morphology, the reduced number of cells in each UB might influence their ability to migrate in the scratch assay. To avoid this potential problem, we also measured the migration of primary UB cells isolated from *Hoxb7/CreGFP;Cfl1^F/F^;Dstn^+/−^* kidneys, which exhibit approximately similar numbers of UB branches as control kidneys at E12.5 (data not shown). The morphology of primary UB cells derived from *Hoxb7/CreGFP;Cfl1^F/F^;Dstn^+/−^* kidneys (Figure 7D–7F) was much better than that of double mutant cells (Figure 6C and 6D), and most of the time they had filled the gap by 24 h after the scratch was made (data not shown). However, their migration rate was slower than that of the wild-type cells: at 8 hr post-scratch, while wild-type cells had filled 61% of the gap, the mutant cells had filled only 31% of the gap (p<0.009) (Figure 7A–7G). Thus, even cells retaining one *Dstn* allele, in the absence of *Cfl1*, have a migration deficit. Altogether, these data suggest that loss of cofilin1 and Destrin in *Cfl1*;*Dstn* double mutants causes actin accumulation and defects in epithelial organization and cell migration, resulting in a failure of branching morphogenesis.

### *Cfl1* is not regulated by GDNF/Ret signaling in kidney

**Figure 8:**
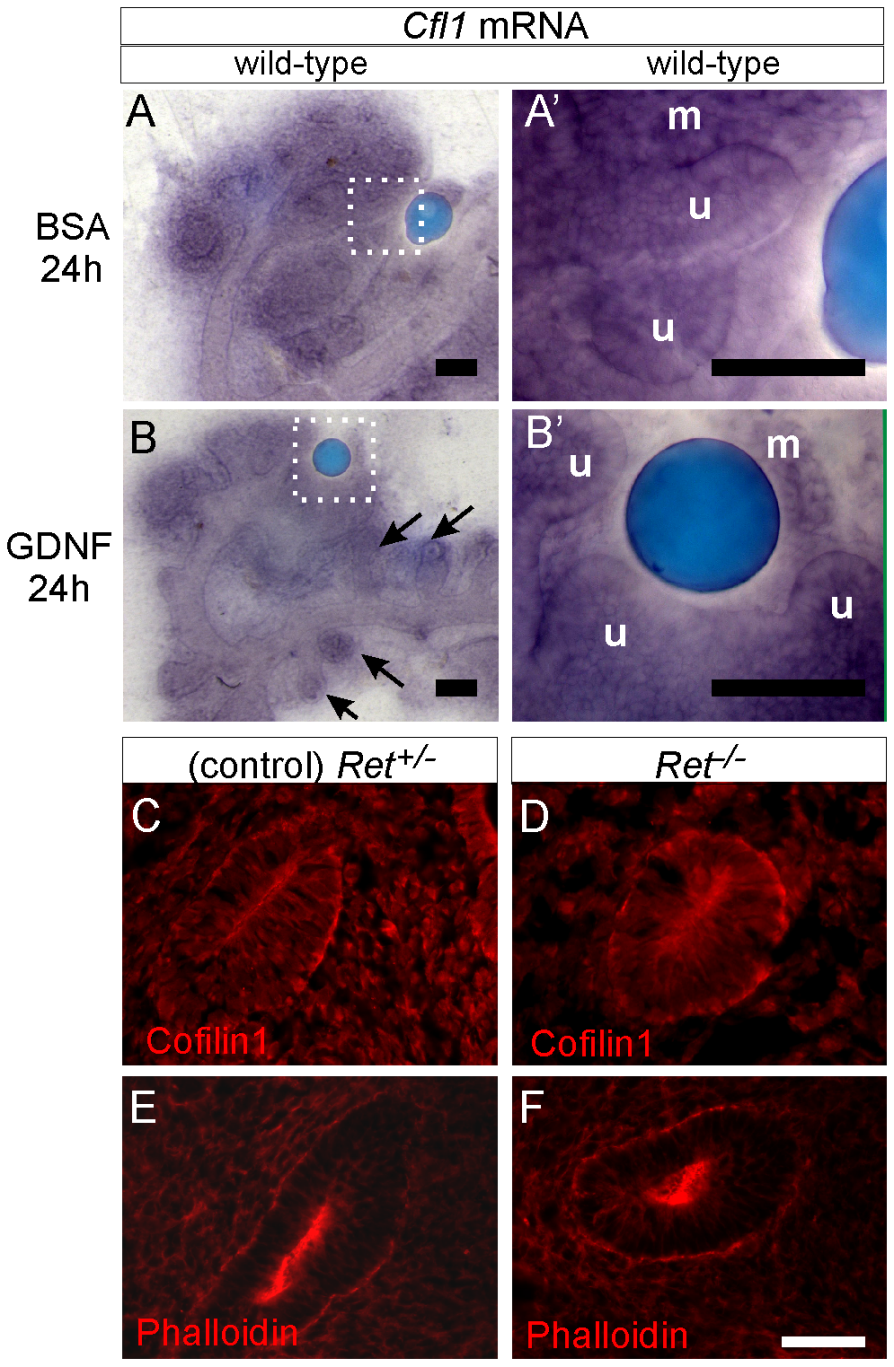
*Cfl1* gene expression is not regulated by GDNF/Ret signaling in developing kidney. (A–A'), *Cfl1 in situ* hybridization in E11.5 wild-type kidney cultured with control, BSA-soaked bead. A' shows a higher magnification of the boxed area in A. mRNA is detected both in metanephric mesenchyme (m) and ureteric epithelium (u). (B–B'), Similar expression levels of *Cfl1* in kidneys cultured with GDNF-soaked beads, which induce enlargement of ureteric tips and extra budding from Wolffian duct (arrows). B' shows a higher magnification of the boxed area in B. (C), Cofilin1 antibody staining of *Ret* ^+/−^ (control) E10.5 embryo, where Wolffian duct shows epithelial thickening as a hallmark of early ureteric bud outgrowth. (D), Normal expression of Cofilin1 protein (red) in *Ret* ^−/−^ Wolffian duct epithelium at E10.5. (E,F) F-actin localization in control and *Ret* ^−/−^ kidneys at E10.5. Scale bars 100 µm for A–B, 50 µm for C–F.

*Cfl1* was identified as one of the potential Ret-induced genes in Ret-expressing NIH3T3 cells [14] and we were therefore interested in determining if *Cfl1* could be induced by Ret signaling in the ureteric epithelium. Both gain- and loss-of-function strategies were used to analyze *Cfl1* mRNA and protein regulation by GDNF/Ret signaling. *Cfl1* is expressed in ureteric epithelium and metanephric mesenchyme of E11.5 kidneys cultured for 24 h (Figure 8A–8A'). While GDNF-soaked beads induced ectopic ureteric budding and local swelling of the UB tip, confirming the functionality of the protein, no change in *Cfl1* mRNA expression was observed (Figure 8B–8B'). Two different *Ret* mutant mouse lines were used to study the effect of either reduced Ret signaling (*Ret*-hypomorphic mice) or lack of Ret signaling (*Ret* ^−/−^ mice) on cofilin1 protein levels. The *Ret*-hypomorphic mutant *Ret^tm2(RET)Vpa^* has reduced UB branching [27] while UB formation fails in most of the *Ret* ^−/−^ kidneys [28]. Cofilin1 was present at normal levels in the early UB of E10.5 *Ret* ^−/−^ mutants (Figure 8C, 8D) as well as in E15 *Ret*-hypomorphic kidneys (data not shown). Furthermore, the distribution of F-actin appeared normal in both types of *Ret*-mutant kidneys (Figure 8E, 8F and data not shown). Therefore, although the failure of UB outgrowth in *Ret* ^−/−^ kidneys is phenotypically similar to the phenotype of *Cfl1;Dstn* double mutant UBs, this is apparently not due to reduced expression of *Cfl1* in the *Ret* mutant.

## Discussion

We examined the requirement for cofilin1- and destrin-mediated cytoskeletal functions during UB branching. While animals retaining at least one wild type *Cfl1* or *Dstn* allele exhibited either normal kidneys or low a frequency of renal/ureteric defects, double homozygotes lacking any cofilin1 or destrin in the UB epithelium had a severe branching defect at an early phase of kidney development. Characterization of cellular defects in *Cfl1*;*Dstn* double mutant animals revealed a huge accumulation of F-actin in UB cells, and a disorganized epithelium, at the same stage when the block in branching occurred. We found that primary UB epithelial cells isolated from *Cfl1;Dstn* double mutants were impaired in their ability to migrate, suggesting that the block in UB growth and branching *in vivo* is due, at least in part, to disruption in normal cell motility.

One important issue that our study addressed was the extent of functional overlap between cofilin1 and destrin, *in vivo*. The biochemical properties of these two proteins are highly similar, but significant functional differences have been observed *in vitro*: for example, destrin is more active in actin-depolymerization, while cofilin1 is a more potent nucleator of actin^ADP^ assembly [29]. These differences, as well as differences in expression patterns, led to the suggestion that the ADFs evolved to fulfill specific requirements for actin filament dynamics in different cell types [18]. During development, the ability of *Cfl1* and *Dstn* to substitute for each other depends on two factors: their overlap in expression and their overlap in function. *Cfl1* is more widely expressed than *Dstn* in the embryo [18], so the ability of *Dstn^−/−^* mice to develop normally (except for corneal defects) could be due to the ability of cofilin1 to replace the absent destrin in many cell types. In *Cfl1^−/−^* mutants, absence of cofilin1 throughout the embryo results in embryonic lethality at E11.5, with specific defects in the neural tube and neural crest cells, even though *Dstn* is strongly upregulated in the *Cfl1^−/−^* embryo [4]. This suggested a specific function for cofilin1. Similarly, the conditional knockout of *Cfl1* in the brain (where *Dstn* is coexpressed) resulted in F-actin accumulation and defects in cell migration and cell cycle progression, indicating that cofilin1 performs roles that cannot be assumed by destrin [5].

In this study, a direct comparison of mice lacking *Cfl1*, *Dstn* or both genes in the UB allowed a clear test of redundancy. The lack of any cellular or developmental defects, or abnormal F-actin accumulation, in the kidneys of *Dstn^−/−^* or *Hoxb7/CreGFP;Cfl1^F/F^* mice suggests that *either* cofilin1 *or* destrin is sufficient for normal cellular functions. Furthermore, when both genes were deleted in the UB cells, there was a complete block to UB growth and branching. This clearly indicates that only in the absence of *Cfl1* is *Dstn* required, while only in the absence of *Dstn* is *Cfl1* required – in other words, there is high degree of functional overlap, at least in this cell lineage.

In embryos in which both *Cfl1* alleles were deleted in the Wolffian duct/ureteric bud lineage, in a *Dstn^−/−^* background, UB growth was arrested shortly after outgrowth from the Wolffian duct, but before further branching. The timing of this developmental block was apparently a consequence of the slow turnover of Cofilin1 protein following deletion of the floxed gene: although the *Hoxb7* promoter is active in the Wolffian duct at least as early as E9.5 [22], [30], and the *Cfl1^F^* alleles were presumably deleted in most or all cells by E10.5, cofilin1 protein was still present at normal levels in the UB at E10.5. Cofilin1 was not absent, nor did excess F-actin accumulate, until ∼E11.5, the approximate stage at which UB branching ceased. Thus, there is no reason to believe that ADF activity has a specific role in UB branching: it likely has a more general role in UB epithelial morphogenesis.

ADFs are important in various cellular processes involving the actin cytoskeleton [1], [31], [32]. We found that simultaneous lack of *Cfl1*;*Dstn* in UB epithelium does not impair the cells' ability to proliferate at normal rates. Therefore, the block in UB branching is not simply due to lack of cell division. Recently it has been demonstrated that cell movements in the Wolffian duct epithelium are important for primary UB outgrowth [10], and it is likely that similar cell movements continue to play a role during later UB growth and branching events. Therefore, the defects in motility of *Cfl1;Dstn* double mutant UB cells, which we observed in a scratch assay using primary UB cell cultures, provides one plausible explanation for the failure of UB growth. In addition to the migratory defects observed in cell cultures, the double mutant UB epithelium *in vivo* displayed unusual heterogeneity in cell size and shape, which is possibly a result of the abnormal accumulation of F-actin preventing the normal re-shaping of epithelial cells during branch formation.

Mice with intermediate numbers of mutant alleles (e.g., with one remaining wild type *Dstn* or *Cfl1* allele) usually developed normal kidneys and ureters, revealing that a single *Dstn* or *Cfl1* gene is usually sufficient, in the UB. But a fraction of these mice displayed visible defects, including double ureter, mild renal hypoplasia or irregular kidney shape. This effect of reduced gene dosage suggests that the level of total ADF expression (cofilin1 + destrin) in UB cells is important, which is consistent with the model, based on biochemical studies, that the various activities of ADFs (severing, stabilizing or nucleating actin filaments) are concentration-dependent [1]. UB cells with only one wild type *Dstn* allele and no *Cfl1* (*Hoxb7/CreGFP*; *Cfl1^F/F^; Dstn^+/−^*) also showed a migration defect in the scratch assay, although less severe than the double-null cells; it is possible that this cellular defect contributes to the phenotypic defects observed in some mice of these genotypes.

Ureter duplications, which occur when the Wolffian duct gives rise to two UBs instead of one, were also seen in a fraction of *Dstn−/−* mice. Ureter duplication can result from excess GDNF/Ret signaling, ectopic GDNF expression, or reduced BMP4, an inhibitor of UB outgrowth [15], but the cause of this defect in ADF-deficient mice is not clear. It has also been observed in embryos lacking the chemokine SDF-1/CXCL12 or its receptor CXCR4 (F.C. and B. Lu, unpublished data), which play an important role in cell migration, and are also known to activate ADF proteins [33]. We therefore asked if *Cxcr4* and *Dstn* would genetically interact in the control of ureteric bud formation, by examining *Dstn* ^−/−^;*Cxcr4* ^−/−^ mutant embryos. These embryos displayed no increase in double UB formation compared to *Dstn^−/−^* alone, failing to support a synergistic role of CXCR4 signaling and ADF activity in this process.

Renal hypoplasia can result from reduced UB growth and branching; while we did not observe a consistent reduction in early UB branching in cultured E11.5 kidneys from embryos of the intermediate genotypes that sometimes cause renal hypoplasia, this may be due to the low penetrance of the defect. The irregular kidney shape sometimes observed in mice of several of the intermediate genotypes is an unusual phenotype, which has not been previously described, to our knowledge. It is likely that this also results from an abnormality in UB growth or elongation, as the collecting duct system is thought to be the main determinant of kidney shape [34]; however, no particular abnormality in early UB morphogenesis was observed in these kidneys, suggesting that the abnormal renal shape arises at a later stage of organogenesis, or else is not revealed in cultured kidneys, which flatten and lose their three dimensional shapes.

One of the most important pathways regulating UB branching is GDNF/Ret signaling, which was previously shown to induce *Cfl1* expression in NIH3T3 cells [14]. The finding that *Cfl1* expression is not regulated by GDNF/Ret signaling in developing kidney was therefore to some extent a surprise, but it is supported by a recent GDNF target screen performed in our laboratory [35], in which no changes in *Cfl1* expression were detected in the UB. We cannot exclude the possibility that activation of Ret signaling would affect ADF protein activity, which is regulated by its phosphorylation/dephosphorylation status [1], but even if this was the case, Ret signaling apparently does not play major role in regulation of actin cytoskeleton dynamics in these cells, as the actin cytoskeleton appeared normal in *Ret*−/− epithelium.

In summary, our results demonstrate that actin depolymerization by the cooperative function of cofilin1 and destrin is essential for normal UB branching, which is blocked due to F-actin accumulation in UB epithelial cells of double mutant *Cfl1;Dstn* mice. As renal abnormalities are common in newborns, this finding may help to understand the origins of certain congenital malformations in humans.

## Materials and Methods

### Ethics statement

All work on animals was conducted under PHS guidelines and approved by the relevant Institutional Animal Care and Use Committees.

### Mouse strains

*Cfl1* null and floxed alleles as well *Ret−/−* and *Ret* hypomorphic (*Ret^tm2(RET)Vpa^*) mice and their genotyping by PCR have been described [4]–[6], [27], [28]. *Dstn^corn1-2J^* [6] mice were genotyped by amplifying the region of genomic DNA where the single mutation occurs with the primers 5′ TCC ACT GCA GCT GTC TTCAGACA 3′ and 5′ ATG ACA AAC CAA TGG ATC CCC AC 3′, then digesting with *BanI*, whose recognition site is mutated, resulting in a Pro106Ser substitution, in *Dstn^corn1-2J^* mice. All mice were on mixed genetic backgrounds (including strains C57BL6/J, FVB/N and 129/SvEv) except for *Ret+/−* mice, which were inbred 129/SvEv.

### Organ cultures and bead experiments

E11.5 or E12.5 kidneys were isolated and cultured on Transwell filters (Fisher) in DMEM with 10% fetal calf serum, 1% Glutamax and 1% penicillin/streptomycin at 37°C and 5% CO_2_ for the indicated times. For GDNF beads, Affigel blue beads (100–200 mesh, Bio-Rad) were washed with PBS/0.1% BSA before incubating with 50 ng/µl recombinant GDNF (R&D) for 30 min at 37°C. Control beads were prepared similarly but incubated in 1% BSA. For organ cultures with GDNF in the culture medium, the concentration was 100 ng/ml.

### Immunofluorescence and measuring mitotic index

Whole mount immunofluorescence staining with anti-Pax2 (1∶200, Zymed) and anti-pan-cytokeratin (1∶200, Sigma) antibodies (AB) was performed as previously described [36]. PFA-fixed 10 µm frozen sections were stained with anti-Calbindin AB (1∶200, Santa Cruz), phosphohistone-H3 (1∶100, Cell Signaling Technology) and phalloidin (1∶40, Molecular Probes). Antigen retrieval by 1 mg/ml pepsin (Sigma) digestion (10 min, 37°C) was performed for the samples stained with Cofilin1 AB [4].

For quantification of mitotic indexes, total epithelial cells and phosphohistone-H3+ epithelial cells were counted in sections through four E11.5 *Dstn^+/−^* and four *Hoxb7/CreGFP; Cfl1^F/F^; Dstn^−/−^* kidneys. For each specimen, the UB cells were counted in 15–19 serial sections. The percentage of pH3+ epithelial cells in mutant and control samples were compared using Student's t-test (two-tailed, equal variance).

### *In situ* hybridization

Samples for whole mount *in situ* hybridization were dipped in ice-cold methanol, and fixed in 4% PFA overnight. Hybridization with digoxigenin-labeled *Cfl1* [4] and *Ret* [37] riboprobes was performed according to Wilkinson [38].

### Non-immortalized primary ureteric bud cultures and scratch assay

After an enzymatic treatment with collagenase (4 µg/µl, Gibco) ureteric buds were dissected free of metanephric mesenchyme and placed on fibronectin coated wells (BD Biosciences) containing DMEM supplemented with 10% FBS, 1% Glutamax, 1% penicillin/streptomycin, 5 ng/ml GDNF, 25 µg/ml FGF2 and 50 µg/ml HGF. Cultures were allowed to settle and form single cell layers for 48 h, before introducing the scratch, using standard 10 µl plastic pipette tips. For immunofluorescence staining, single cell layers were fixed in 4% PFA for 10 min, washed with PBS, and incubated with anti-pan-cytokeratin (1∶200, Sigma) and anti-Ki67 (1∶100, Abcam) antibodies or with phalloidin (1∶40, Molecular Probes). To quantify the cell migration, the width of the gap formed by the scratch was measured immediately after the scratch, and 3 h and 8 h later. The proportion of gap filled was calculated by dividing the width at each time by the initial gap width. All measurements were done using Image J program.

## Supporting Information

Figure S1 Double ureters in *Dstn-/-* and delayed UB branching in *Cfl1^+/-^;Dstn^-/-^* embryos. (A-B), double ureter (arrows) in a *Dstn^-/-^* embryo (B) compared to single ureter in control (A), at E11.5. (C-F), *Hoxb7/myrVenus* transgene expressed in ureteric bud epithelium reveals slightly delayed branching morphogenesis in a *Cfl1^+/-^;Dstn^-/-^* kidney at E12.5. C and E, *Dstn^+/-^* (control) kidney cultured for 18h (C) and 36h (E). (D) and (F), *Cfl1^+/-^;Dstn^-/-^* kidney has fewer ureteric tips at 18h (D) and shows delayed branching at 36h (F). Scale bar 200 μm.
(1.62 MB TIF)

Figure S2 Normal expression of *Ret* and *Wnt11* in *Cfl1;Dstn* double mutant UBs. (A-D) Control and double mutant kidneys at E11.5 (A, B) or E11.5+18hrs of culture (C, D) were used for Ret whole mount *in situ* hybridization. (A) control (no Cre; *Cfl1^F/+^; Dstn^-/-^*), (B) double mutant. The UBs are demarcated by dotted lines. (C) control (*Hoxb7/CreGFP; Cfl1^+/+^; Dstn^+/-^*), (D) double mutant. *Ret* expression in double mutants is initially normal (B) but reduced after 18 hours culture (D). The localization of transcripts at the UB tip remains normal (arrows). (E-F) Control (E, no Cre; *Cfl1^F/F^; Dstn^+/-^*) and double mutant (F) kidneys at E11.5 were used for Wnt11 whole mount *in situ* hybridization. The UBs are demarcated by dotted lines. Wnt11 expression is normal in the double mutant UB tip.
(1.77 MB TIF)

Figure S3 F-actin distribution is normal in double mutant Wolffian duct at E10.5. Embryos were stained for phalloidin (red) to visualize F-actin and for calbindin (green) to demarcate the Wolffian duct and ureteric bud epithelium. (A), Wild type embryo. (A') shows higher magnification of F-actin distribution in wild type Wolffian duct. F-actin remains normal in (B) *Hoxb7/CreGFP;Cfl1^F/F^;Dstn^+/-^* and (C) *Hoxb7/CreGFP;Cfl1^F/F^;Dstn^-/-^* Wolffian ducts. (B') and (C') are enlargements of epithelium in (B) and (C), respectively. Scale bar; for (A-C) 100 μm; for (A'-C') 50 μm.
(2.60 MB TIF)

Figure S4 Establishment of primary UB cell cultures. Ureteric buds were isolated free of metanephric mesenchyme and plated in fibronectin coated wells, where they attach and form monolayers. (A), Primary ureteric epithelial cells of all genotypes (*Dstn^+/-^* is shown) were relatively quiescent in culture, as shown by the paucity of Ki67+ (green) proliferative cells. Phalloidin stain for F-actin is in red. (B), Pan-cytokeratin (green) staining indicates that cells in culture are ureteric epithelium-derived (as shown in a Hoxb7/CreGFP; Cfl1F/F culture, as an example), while phalloidin (red) visualizes F-actin. (C-D), Primary ureteric epithelial cell cultures from (A) control (*Dstn^+/-^*) and (B) double mutant kidneys, 20h after plating. No difference was observed between the ability of control and double mutant cells to form these monolayers.
(2.72 MB TIF)
