# Supplementary figures and images for: Actin Depolymerizing Factors Cofilin1 and Destrin Are Required for Ureteric Bud Branching Morphogenesis
Source: PLoS Genet. 2010 Oct 28;6(10):e1001176. doi: 10.1371/journal.pgen.1001176 (PMC2965756; doi:10.1371/journal.pgen.1001176)

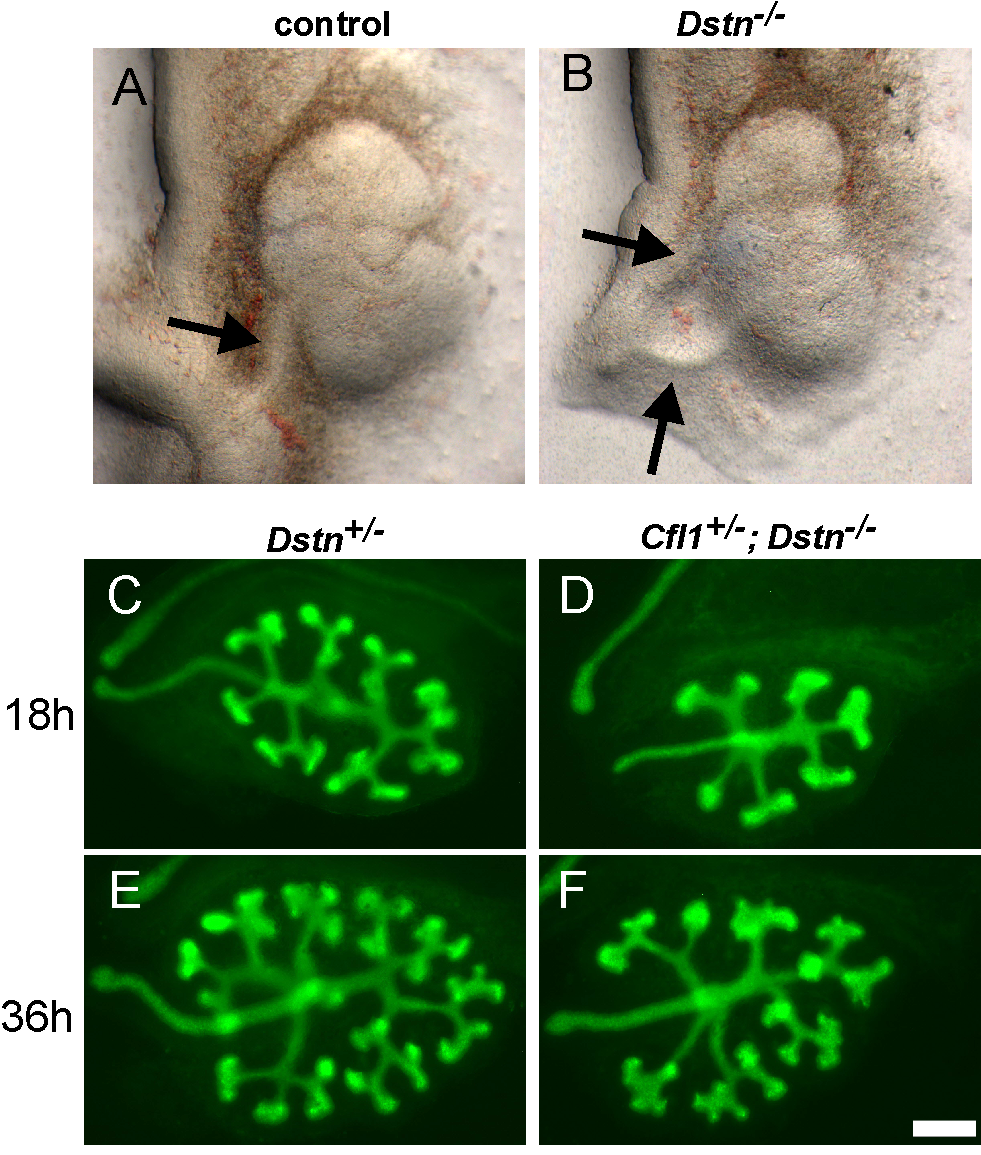

Supplement: Figure S1 — Double ureters in Dstn-/- and delayed UB branching in Cfl1+/-;Dstn-/- embryos. (A-B), double ureter (arrows) in a Dstn-/- embryo (B) compared to single ureter in control (A), at E11.5. (C-F), Hoxb7/myrVenus transgene expressed in ureteric bud epithelium reveals slightly delayed branching morphogenesis in a Cfl1+/-;Dstn-/- kidney at E12.5. C and E, Dstn+/- (control) kidney cultured for 18h (C) and 36h (E). (D) and (F), Cfl1+/-;Dstn-/- kidney has fewer ureteric tips at 18h (D) and shows delayed branching at 36h (F). Scale bar 200 μm. (1.62 MB TIF) [file pgen.1001176.s001.tif]

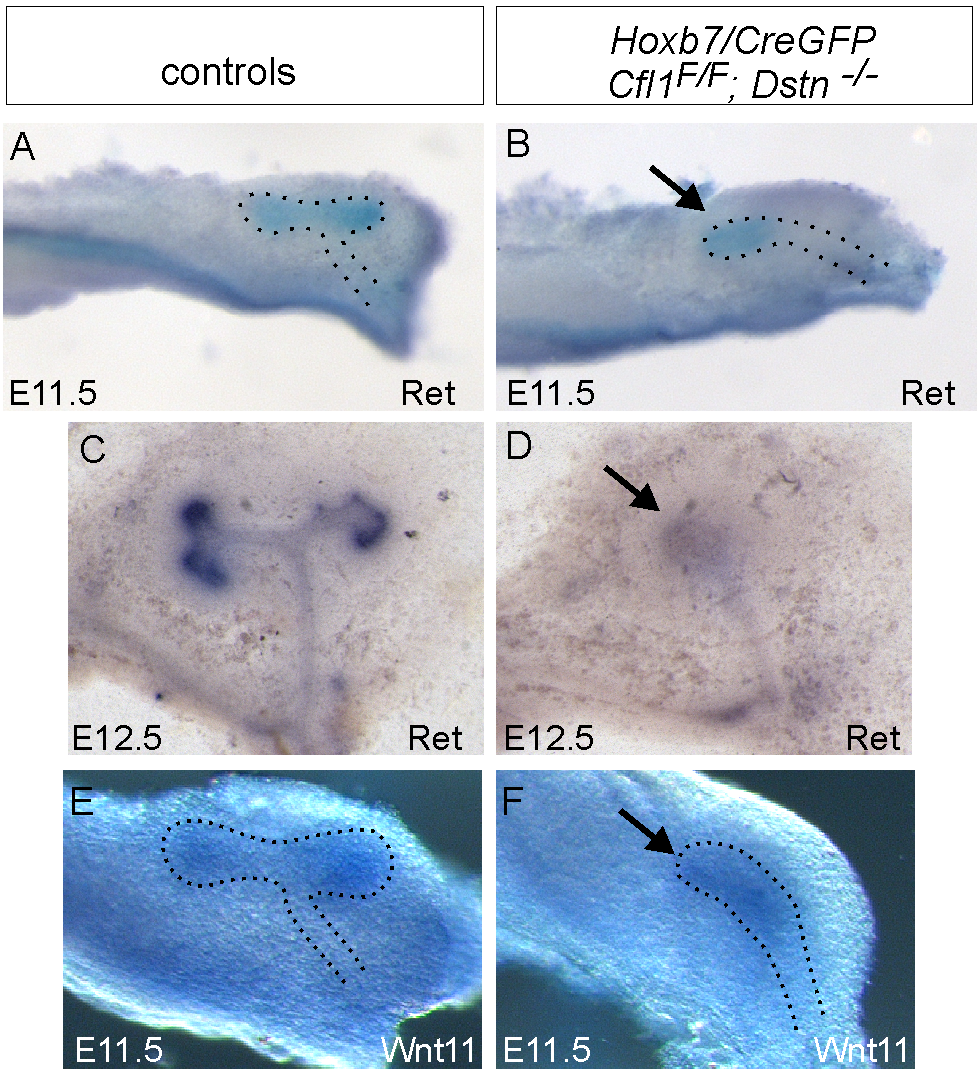

Supplement: Figure S2 — Normal expression of Ret and Wnt11 in Cfl1;Dstn double mutant UBs. (A-D) Control and double mutant kidneys at E11.5 (A, B) or E11.5+18hrs of culture (C, D) were used for Ret whole mount in situ hybridization. (A) control (no Cre; Cfl1F/+; Dstn-/-), (B) double mutant. The UBs are demarcated by dotted lines. (C) control (Hoxb7/CreGFP; Cfl1+/+; Dstn+/-), (D) double mutant. Ret expression in double mutants is initially normal (B) but reduced after 18 hours culture (D). The localization of transcripts at the UB tip remains normal (arrows). (E-F) Control (E, no Cre; Cfl1F/F; Dstn+/-) and double mutant (F) kidneys at E11.5 were used for Wnt11 whole mount in situ hybridization. The UBs are demarcated by dotted lines. Wnt11 expression is normal in the double mutant UB tip. (1.77 MB TIF) [file pgen.1001176.s002.tif]

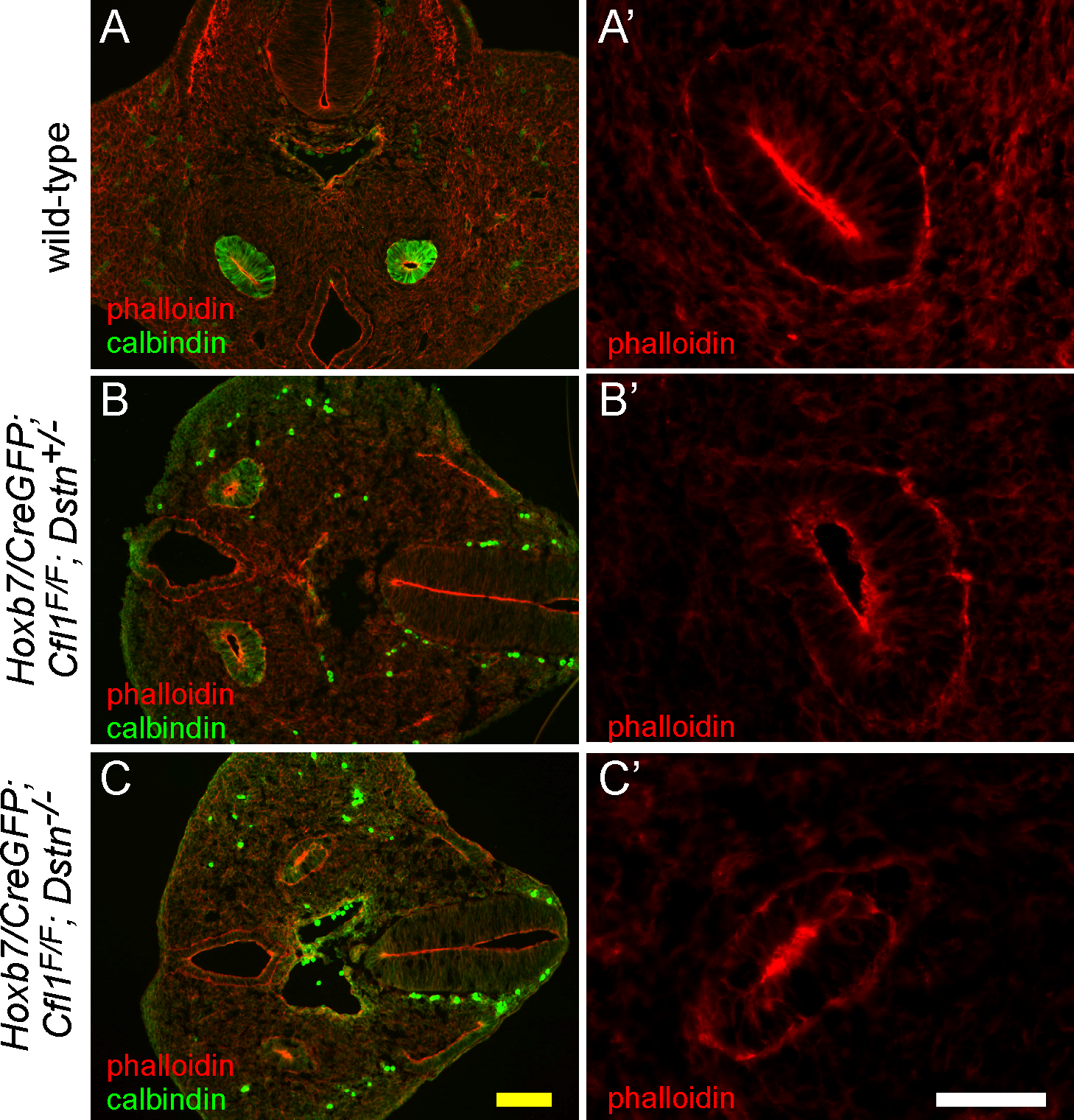

Supplement: Figure S3 — F-actin distribution is normal in double mutant Wolffian duct at E10.5. Embryos were stained for phalloidin (red) to visualize F-actin and for calbindin (green) to demarcate the Wolffian duct and ureteric bud epithelium. (A), Wild type embryo. (A') shows higher magnification of F-actin distribution in wild type Wolffian duct. F-actin remains normal in (B) Hoxb7/CreGFP;Cfl1F/F;Dstn+/- and (C) Hoxb7/CreGFP;Cfl1F/F;Dstn-/- Wolffian ducts. (B') and (C') are enlargements of epithelium in (B) and (C), respectively. Scale bar; for (A-C) 100 μm; for (A'-C') 50 μm. (2.60 MB TIF) [file pgen.1001176.s003.tif]

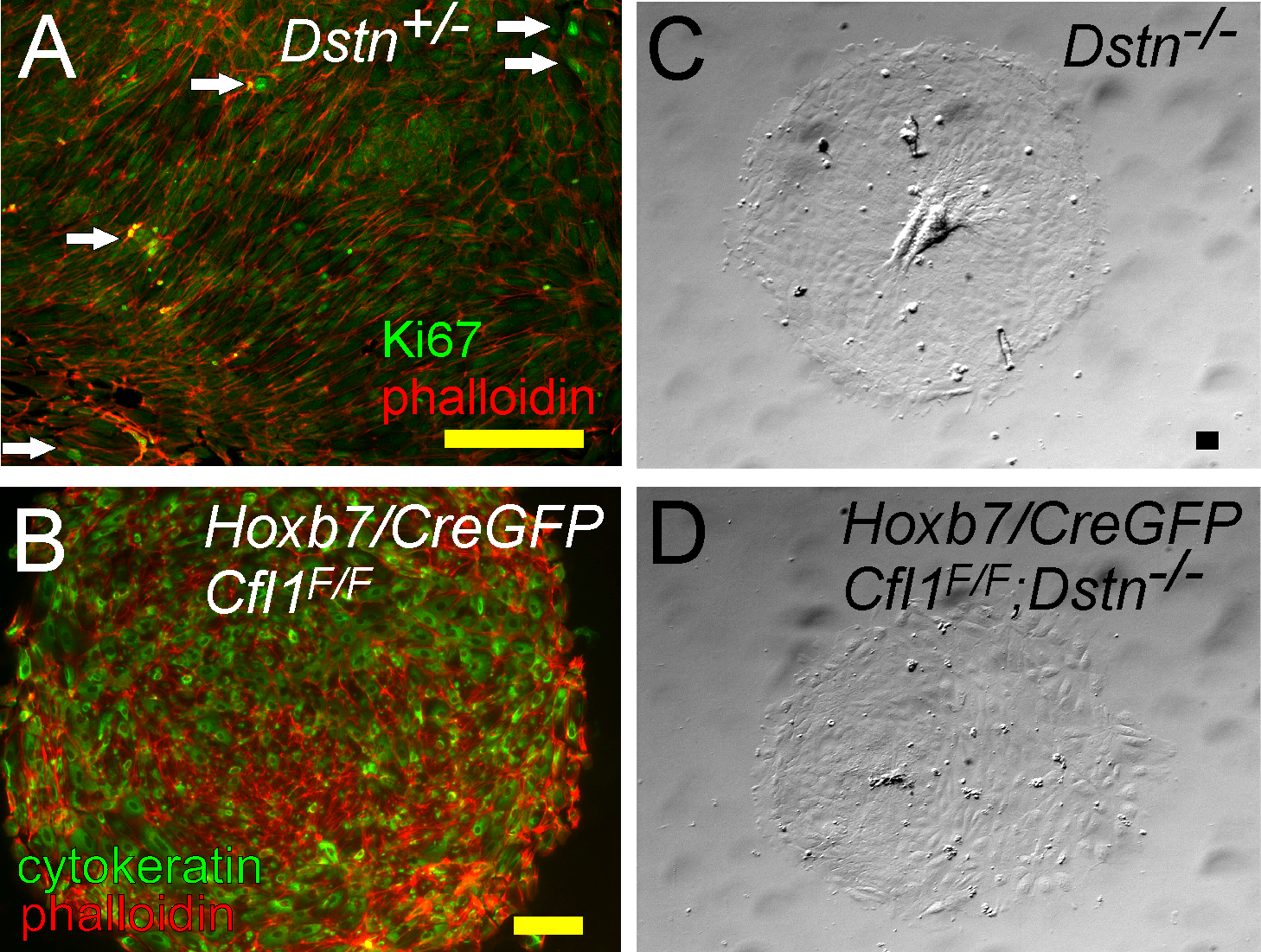

Supplement: Figure S4 — Establishment of primary UB cell cultures. Ureteric buds were isolated free of metanephric mesenchyme and plated in fibronectin coated wells, where they attach and form monolayers. (A), Primary ureteric epithelial cells of all genotypes (Dstn+/- is shown) were relatively quiescent in culture, as shown by the paucity of Ki67+ (green) proliferative cells. Phalloidin stain for F-actin is in red. (B), Pan-cytokeratin (green) staining indicates that cells in culture are ureteric epithelium-derived (as shown in a Hoxb7/CreGFP; Cfl1F/F culture, as an example), while phalloidin (red) visualizes F-actin. (C-D), Primary ureteric epithelial cell cultures from (A) control (Dstn+/-) and (B) double mutant kidneys, 20h after plating. No difference was observed between the ability of control and double mutant cells to form these monolayers. (2.72 MB TIF) [file pgen.1001176.s004.tif]
